# Enquiring About Tolerance (EAT) study: Feasibility of an early allergenic food introduction regimen

**DOI:** 10.1016/j.jaci.2015.12.1322

**Published:** 2016-05

**Authors:** Michael R. Perkin, Kirsty Logan, Tom Marrs, Suzana Radulovic, Joanna Craven, Carsten Flohr, Gideon Lack, EAT Study Team, Louise Young, Victoria Offord, Mary DeSousa, Jason Cullen, Katherine Taylor, Anna Tseng, Bunmi Raji, Sarah Nesbeth, Gillian Regis, Charlie Bigwood, Charlotte Stedman, Sharon Tonner, Emily Banks, Yasmin Kahnum, Rachel Babic, Ben Stockwell, Erin Thompson, Lorna Wheatley, Devi Patkunam, Kerry Richards, Ewa Pietraszewicz, Alick Stephens, Asha Sudra, Victor Turcanu

**Affiliations:** a Population Health Research Institute, St George's, University of London, London, United Kingdom; b Department Paediatric Allergy, Division of Asthma, Allergy and Lung Biology, King's College London, London, United Kingdom; c St John's Institute of Dermatology, Guy' and St Thomas' Hospital NHS Foundation Trust, London, United Kingdom

**Keywords:** Food allergy, diet, allergens, infancy, breastfeeding, EAT, Enquiring About Tolerance, EIG, Early introduction group, IFS2010, Infant Feeding Survey 2010, LEAP, Learning Early About Peanut Allergy, SIG, Standard introduction group, SPT, Skin prick test, UK, United Kingdom

## Abstract

**Background:**

The influence of early exposure to allergenic foods on the subsequent development of food allergy remains uncertain.

**Objective:**

We sought to determine the feasibility of the early introduction of multiple allergenic foods to exclusively breast-fed infants from 3 months of age and the effect on breastfeeding performance.

**Methods:**

We performed a randomized controlled trial. The early introduction group (EIG) continued breastfeeding with sequential introduction of 6 allergenic foods: cow's milk, peanut, hard-boiled hen's egg, sesame, whitefish (cod), and wheat; the standard introduction group followed the UK infant feeding recommendations of exclusive breastfeeding for around 6 months with no introduction of allergenic foods before 6 months of age.

**Results:**

One thousand three hundred three infants were enrolled. By 5 months of age, the median frequency of consumption of all 6 foods was 2 to 3 times per week for every food in the EIG and no consumption for every food in the standard introduction group (*P* < .001 for every comparison). By 6 months of age, nonintroduction of the allergenic foods in the EIG was less than 5% for each of the 6 foods. Achievement of the stringent per-protocol consumption target for the EIG proved more difficult (42% of evaluable EIG participants). Breastfeeding rates in both groups significantly exceeded UK government data for equivalent mothers (*P* < .001 at 6 and at 9 months of age).

**Conclusion:**

Early introduction, before 6 months of age, of at least some amount of multiple allergenic foods appears achievable and did not affect breastfeeding. This has important implications for the evaluation of food allergy prevention strategies.

*Discuss this article on the JACI Journal Club blog:* *www.jaci-online.blogspot.com*.

The point prevalence of self-reported food allergy in a recent systematic review was around 6%,^1^ and that for particular foods is increasing.^2^ The role of allergen consumption in early infancy and its effect on the development of allergy or tolerance to food proteins remains uncertain.

The World Health Organization Global Strategy for Infant and Young Child Feeding,^3^ which is endorsed by the United Kingdom (UK) Government,^4^ recommends exclusive breastfeeding for the first 6 months with nutritious complementary foods introduced thereafter and continued breastfeeding up to the age of 2 years or beyond.^5^ The UK Government infant feeding information leaflet for parents, “Weaning—starting solid food,” adopts a more pragmatic target of *around* 6 months of exclusive breast-feeding.^6^ It also states that if a mother decides to introduce complementary foods before 6 months of age, there are some foods that should be avoided because they can cause allergies, including “wheat-based foods...eggs, fish, shellfish, nuts (and) seeds.” There is little evidence that this reduces allergic disease.^7^ Interventions involving maternal diet during pregnancy alone^8^ or pregnancy and lactation^9^ and alterations to the timing and type of solid food introduction in infants^10^ have thus far not halted the increase in food allergy. Furthermore, there is now observational evidence that early introduction of cow's milk,^11^ peanut,^12^ or egg^13^ during infancy might prevent the development of food allergies.

In 2010, the UK government published the latest of its quinquennial reviews of infant feeding practice in the country (Infant Feeding Survey 2010 [IFS2010]).^14^ Although the UK Government guidelines no longer stipulate delaying the introduction of allergenic foods beyond 6 months of age, the current feeding regimen of UK mothers clearly does delay introduction. At 8 to 10 months of age, only 8% of infants had been given peanuts or peanut products.^14^

The significant trend toward later introduction of solid foods and longer duration of exclusive breastfeeding in the UK has coincided with the prevalence of food allergy appearing to increase.^15^ Although delayed introduction of allergenic foods prevents occurrence of an allergic reaction, there is no evidence to suggest it prevents the development of allergies and might simply delay the manifestation of a pre-existing allergy.

The Solids Timing for Allergy Research study suggested that induction of immune tolerance pathways is possible through early introduction of egg and resulted in a reduction, although a nonsignificant one, in egg allergy incidence.^16^ The Learning Early About Peanut Allergy (LEAP) study found that early introduction of peanut into the diets of high-risk atopic infants protects against the development of peanut allergy.^17^, ^18^

The Enquiring About Tolerance (EAT) study has a wider remit, namely to test the hypothesis that the early introduction of multiple allergenic foods from 3 months of age in an unselected population of exclusively breastfed infants will, as a primary outcome, reduce the prevalence of food allergy and, as a secondary outcome, influence asthma, eczema, allergic rhinitis, and the prevalence of combined allergic disease by 3 years of age.

The EAT study has completed enrollment with 1303 participants. All participants are now beyond 2 years of age, and this milestone affords the opportunity to present the study methodology and assess the feasibility and acceptability of the introduction regimen in this unique cohort.

## Methods

The EAT study is a population-based randomized controlled trial that enrolled exclusively breastfed infants from England and Wales regardless of atopic status or family history of allergy. Infants who had consumed anything other than breast milk or water since birth, were part of multiple births, were born prematurely, had any serious medical condition, or were participating in other medical research were not eligible for enrollment. A current household member with a food allergy was not an exclusion criterion.

Ethical approval for the EAT study was provided by St Thomas' Hospital REC (REC reference 08/H0802/93), and the study is registered with the International Standard Randomized Controlled Trial Number Register (14254740). Informed consent was obtained from the parents of all children enrolled in the study, and safety data were regularly reviewed by the EAT study's independent data monitoring committee.

**Fig 1:**
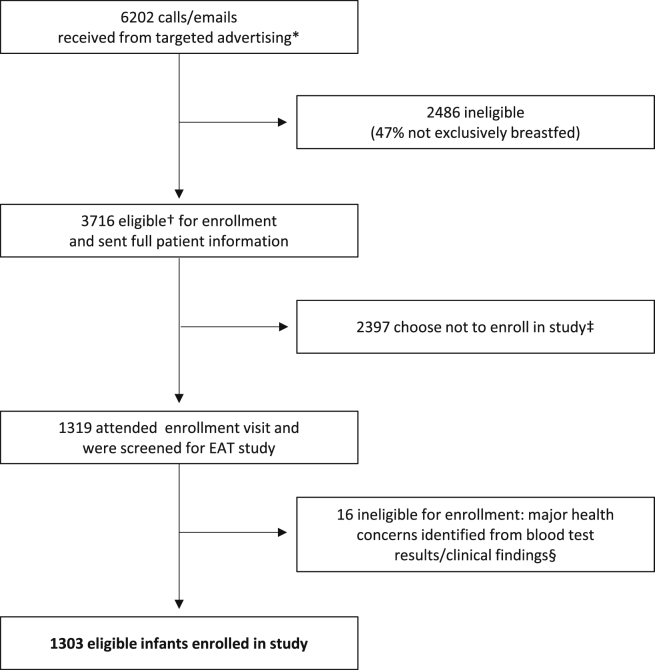
EAT study recruitment. *Direct mailing of families with infants aged 5 to 11 weeks in England and Wales. †Exclusively breastfed at enrollment, 37 or more weeks' gestation, singleton birth, no parental report of major health concerns, not taking part in other research, willing to attend 3 study visits over a 3-year period, willing to be randomized to either study group, and not planning to move from the UK for the study's duration. ‡Reasons included concerns about participation requirements on reading of the full patient information sheet, wanting to have more flexibility with early feeding, concerns about traveling to London, child's father not happy with participation, unable to reach enrollment visit without introducing formula and/or solid food, and too many other commitments. §Eight infants randomized to each group were found to have significant health issues either on blood testing or the clinical examination at the enrollment visit rendering them ineligible for enrollment: conditions included severe vitamin D deficiency, severe iron deficiency, severe failure to thrive, familial hypercholesterolemia, congenital stridor, epidermolysis bullosa, and cartilage hair hypoplasia syndrome.

Families were recruited to the study from those who responded to a flyer mailed to parents of young infants throughout England and Wales (Fig 1). The 6 allergenic foods selected to form the trial's intervention, cow's milk, peanut, hen's egg, sesame, whitefish (cod), and wheat, were chosen from the foods most commonly found to be responsible for IgE-mediated food reactions in children.^19^, ^20^ The trial's primary outcome is the prevalence of IgE-mediated food allergy, which we aimed to confirm using double-blind, placebo-controlled food challenge to 1 or more of the 6 intervention foods at between 1 and 3 years of age (see Table E1 in this article's Online Repository at www.jacionline.org). The trial is powered at 80% to detect a halving of food allergy prevalence between the study groups. At study commencement, the expected food allergy prevalence in the standard introduction group (SIG) was 6%. An analysis undertaken after 3 months of recruitment indicated that the EAT parental atopy rate was higher than that of a contemporary UK population-based study.^21^ Data from the Early Prevention of Asthma in Atopic Children study was used to extrapolate the expected SIG food allergy rate based on the observed prevalence of 30% visible eczema among these initial participants.^22^ Taken together, the revised estimate of expected food allergy prevalence in the SIG group was 8%. A principle intention-to-treat analysis will be undertaken for children evaluable for the primary outcome, with a secondary per-protocol analysis assessing the effect of degree of compliance on the primary outcome.

### Trial design

**Fig 2:**
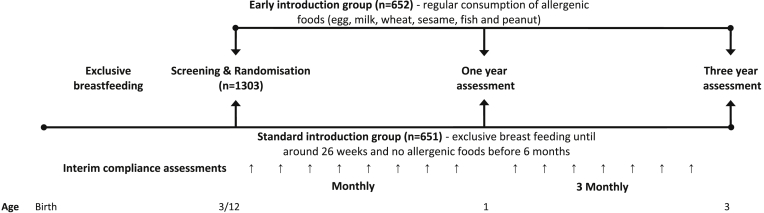
EAT study overview.

Between 13 and 17 weeks of age, enrolled infants were randomly assigned to either the SIG or the early introduction group (EIG). Fig 2 shows the overall EAT study design.

### SIG

Those randomized to the SIG were asked to comply completely with the current UK government infant feeding guidelines of exclusive breastfeeding until around 6 months of age and no consumption of allergenic foods before 6 months of age. After 6 months of age, introduction of allergenic foods was left to parental discretion.

### EIG

Infants in the EIG were randomized to the sequential introduction of the 6 chosen allergenic foods alongside continued breastfeeding (see Fig E4 in this article's Online Repository at www.jacionline.org). Infants in this group underwent skin prick tests (SPTs) in duplicate to the 6 intervention foods and an open incremental food challenge if they showed any sensitization (SPT response >0 mm, no upper limit). Children who were not sensitized or who were sensitized but had a subsequent negative food challenge result were asked to follow the EIG introduction regimen. Those given a diagnosis of allergy based on results of a food challenge were advised to avoid that food and continue the introduction regimen for the other allergenic foods. Fundamental to the trial design was the intention that breast milk should remain an important source of nutrition until at least 6 months of age, regardless of study group. The EIG introduction regimen is described in more detail in the Methods section in this article's Online Repository at www.jacionline.org.

### Online interim questionnaires

An online questionnaire completed monthly until 12 months of age and every 3 months between 12 and 36 months of age by the infants' parents was the main portal of communicating information about the health and diet of the participants to the study team. Parents reported any atopic symptoms in their children and any adverse events (serious and nonserious) through the online questionnaire.

### Consumption monitoring

**Fig E1:**
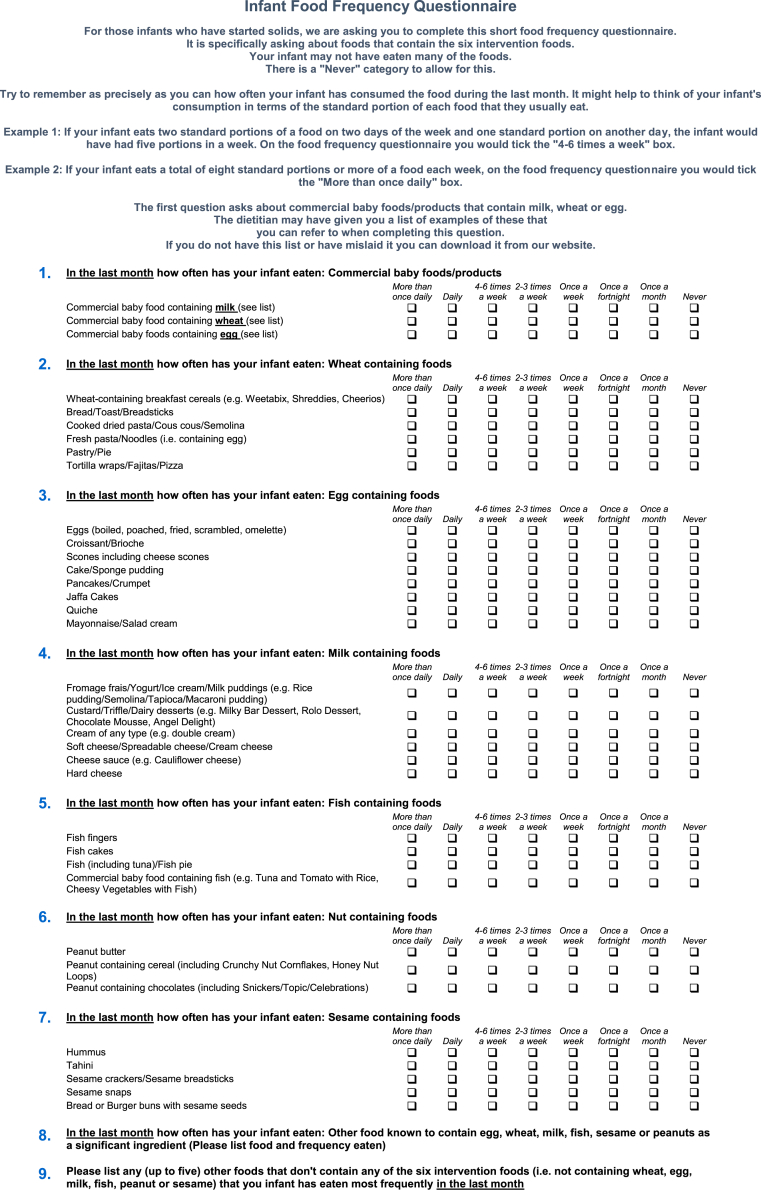
EAT study food frequency questionnaire.

Within this online questionnaire, both groups completed a food frequency questionnaire section assessing how frequently foods containing the 6 study allergens were being consumed (see Fig E1 in this article's Online Repository at www.jacionline.org).

EIG families kept a weekly diary until 1 year of age and monthly thereafter to assess the degree to which they were meeting the consumption target of 4 g of each allergenic food protein per week. For each of the last 4 complete weeks preceding the child's monthly birthday and for each of the allergenic foods, parents recorded the percentage of the recommended amount of food their child was consuming (100%, 75%, 50%, ≤25%, or not tried yet), with guidance provided on the amount of each food constituting those percentages. These diary data were then entered into the online questionnaires.

### Per-protocol compliance: Overall and food specific

The overall per-protocol compliance criteria for the SIG and EIG are listed in Table I. Further information about how the responses from the online questionnaires were used to determine whether per-protocol compliance was assessable for each participant and whether the criteria in Table I had been fulfilled in each group is explained in more detail in the Methods section in this article's Online Repository.

### Holistic assessment

Participants in the study undergo a comprehensive series of investigations aimed at understanding what causes sensitization and food allergy to emerge in children (see the Methods section in this article's Online Repository).

## Results

The EAT study recruited a cohort of 1303 three-month-old infants who were both geographically and demographically representative of the population of England and Wales (Table II).^14^, ^21^, ^23^, ^24^, ^25^, ^26^, ^27^ The prevalence of visible eczema at the 3-month enrollment visit was 24.4%, and filaggrin mutation carriage was 11.9%. Parental history of atopy (any eczema, asthma, or hay fever in either parent) affected 81.9% of the cohort.

Prevalence of sensitization (SPT >0 mm) in the EIG at the enrollment visit was 5.1% (33/652). Nine children were sensitized to cow's milk (SPT range, 2.5-7 mm), 9 to peanut (SPT range, 1-4 mm), 24 to egg (SPT range, 1.75-16 mm), none to sesame, 1 to cod (SPT range, 2.75 mm), and 2 to wheat (SPT range, 1.5-2.25 mm). Eight children were sensitized to 2 or more foods (milk/egg, 3 infants; milk/peanut, 2 infants; egg/cod, 1 infant; peanut/egg/wheat, 1 infant; and milk/peanut/egg, 1 infant). Histamine, like the food allergens, was tested in duplicate. There were no children with double-negative histamine responses.

### Breastfeeding in the EAT study

**Fig 3:**
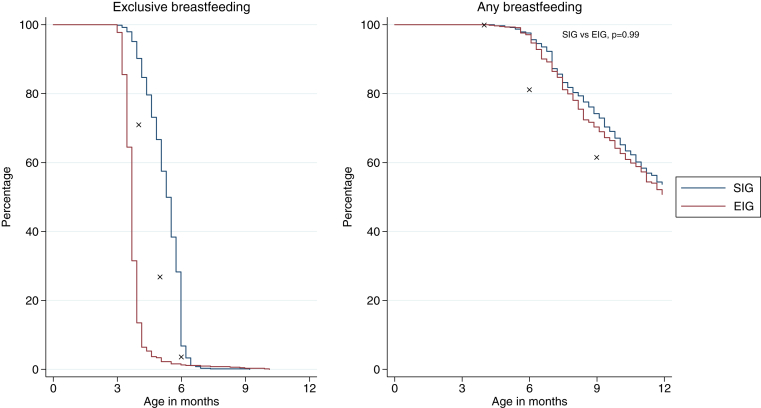
Breastfeeding in the EAT cohort. ✘, IFS2010 data. All comparisons between EIG or SIG and IFS2010 data at varying ages were statistically significant (*P* < .001). Data are available for exclusive breastfeeding (SIG, 633 [97.2%]; EIG, 622 [95.4%]) and any breastfeeding (SIG, 620 [95.2%]; EIG, 583 [89.4%]).

The EAT study aimed to maintain high breastfeeding rates in the EIG and achieve high levels of exclusive breastfeeding in the SIG, in line with UK Government infant feeding policy. For the EIG, the intention was that exclusive breastfeeding ceased with the introduction of baby rice (or something similar) shortly after enrollment. In the EIG 97% (593/610) of infants were still being breastfed alongside solid food consumption at 6 months of age. This is significantly higher than the 81% reported to be breastfeeding at 6 months of age by IFS2010 (*P* < .001) among those mothers who had breastfed to 4 months of age (Fig 3).

In the SIG 67% (425/636) of infants were still being exclusively breastfed at 5 months of age versus 27% in the IFS2010 by using the baseline of IFS2010 infants exclusively breastfed at 3 months of age (*P* < .001). At 6 months of age, 29% (137/636) of infants were still being exclusively breastfed compared with only 4% in the equivalent IFS2010 infants (*P* < .001). Similar to the EIG, 98% (618/633) of mothers in the SIG were still breastfeeding by the time their child was 6 months of age (Fig 3).

### Allergenic food consumption

**Fig 4:**
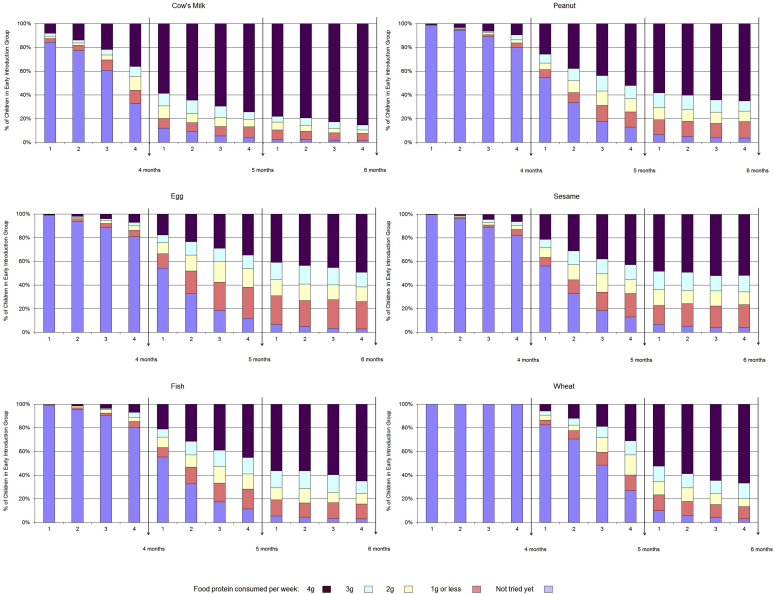
Consumption of allergenic foods by the EIG in the 4 weeks before their 4-, 5-, and 6-month birthdays. Data were available for 4 (581 [89.1%]), 5 (548 [84.0%]), and 6 (537 [82.4%]) months.

Allergenic food consumption in the EIG from enrollment to 6 months of age is presented in Fig 4. The data are taken from the 4-, 5-, and 6-month online questionnaires and refer to the 4 weeks previous to the participant's monthly birthday. Questionnaire completion rates were high (EIG: 90% at 4 months and 84% at 5 and 6 months). By 6 months, consumption of each allergenic food had occurred in more than 95% of EIG infants (Fig 4). The quantity of allergenic food consumed and the speed of introduction varied for each food. The protocol introduced cow's milk (as yogurt) as the first allergenic food, and this also being a familiar infant food was reflected in the consumption results. Wheat was introduced last and not before 4 months of age, and adherence to this instruction was 100%. The proportion of EIG infants consuming the recommended amount of 4 g of food protein per week by 6 months of age was as follows: cow's milk, 85%; peanut, whitefish, and wheat, 65%; and egg and sesame, 50% (Fig 4).

**Fig 5:**
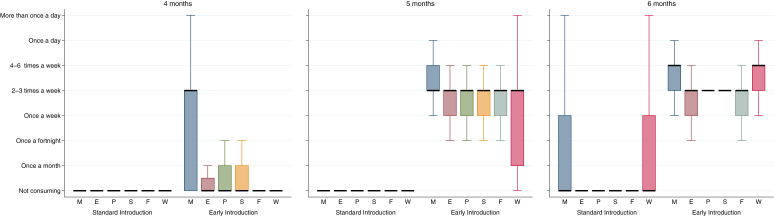
Differences in frequency of allergenic food consumption in the SIG and EIG by 4, 5, and 6 months of age. Data were available for 4 (SIG, 621 [95.4%]; EIG, 588 [90.2%]), 5 (SIG, 612 [94.0%]; EIG, 550 [84.4%]), and 6 (SIG, 605 [92.9%]; EIG, 542 [83.1%]) months. *Black bar*, Median; *box upper hinge*, 75th percentile; *box lower hinge*, 25th percentile; *upper whisker*, upper quartile + 1.5 * interquartile range; *lower whisker*, lower quartile − 1.5 * interquartile range. *P* < .0005, SIG versus EIG for every individual food at every time point.

Cow's milk formula introduction was minimal in both groups before 6 months of age: 2% in the SIG and 3% in the EIG ever having had cow's milk formula by 4 months of age and 7% in both groups ever having had cow's milk formula by 5 months of age. It was unknown whether mothers would adhere to the SIG regimen and avoid early introduction of the allergenic foods. Fig 5 shows the differences between frequency of consumption of allergenic foods in the SIG and EIG at 4, 5, and 6 months of age. For every allergenic food, in each age group there was significantly higher consumption in the EIG than the SIG (*P* < .001 for each food). There was minimal consumption of all allergenic foods in the SIG until 6 months of age, when there was an increase in consumption of milk and wheat, although these were still consumed significantly less frequently than in the EIG (*P* < .0005). Only 2.6% of evaluable SIG participants had introduced any peanut, egg, sesame, fish, or wheat before 5 months of age (Table I, criterion C). By 6 months of age, 5.6% of evaluable SIG participants had been given cow's milk formula in a volume exceeding 300 mL for 1 day or more (Table I, criterion D), 8.8% had been given less than 300 mL/d, and 85.6% had never had any cow's milk formula.

In the EIG consumption was low for all allergenic foods except milk at 4 months of age but increased to a median of at least twice-weekly consumption for all allergenic foods at 5 and 6 months of age. However, although the median frequency of consumption of the 6 allergenic foods was at least twice weekly at 5 and 6 months of age, 4 of the 6 foods (peanut, egg, sesame, and whitefish) at 5 months and 2 (egg and whitefish) at 6 months of age were being consumed by 25% of EIG participants only once a week.

### Overall per-protocol compliance

For more information on overall per-protocol compliance, see Table I. The combination of the enhanced difficulty of being compliance assessable in the EIG (see section on per-protocol compliance status appraisal in the Methods section in this article's Online Repository) and a lower questionnaire completion rate in the EIG (data not shown) meant that there was a difference in the proportion whose compliance status was nonevaluable between the 2 groups (SIG, 7%; EIG, 19%). Hence participants in both groups in the EAT study fell into 3 compliance categories: compliant, noncompliant, and compliance not evaluable (not having completed the requisite questionnaires or formal dropouts from the study).

Ninety-two percent (558/606) of compliance-evaluable children in the SIG met the definition of per-protocol compliance (Table I). Forty-two percent (223/529) of compliance-evaluable EIG children complied entirely with the protocol and consumed 3 g or more of the allergenic food protein for 5 of more of the intervention foods for 5 or more weeks between 3 and 6 months of age (Table I). These figures represent 86% (558/651) and 34% (223/652) of the whole SIG and EIG groups, respectively.

**Fig E2:**
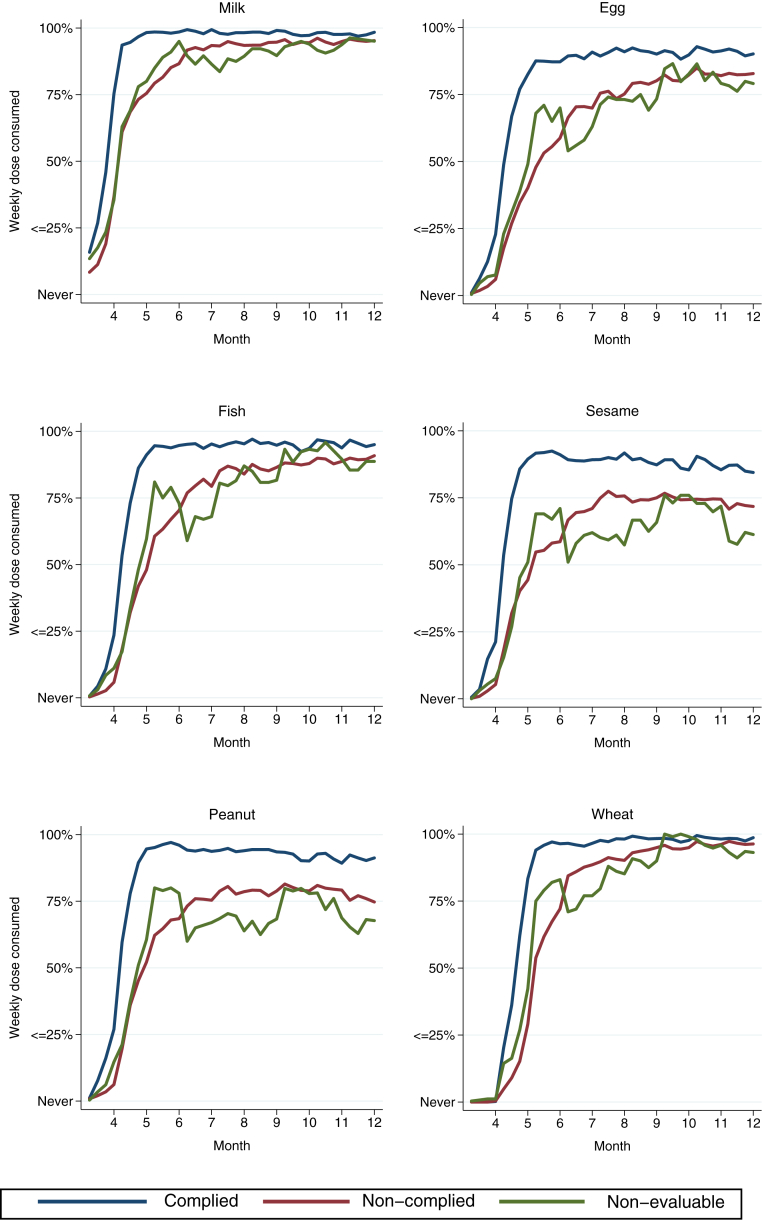
EIG compliance status and early introduction of allergenic foods. The mean dose of allergenic protein consumed each week from enrollment to 12 months of age for each allergenic food is presented for the EIG group. The EIG group is divided into those who were overall compliant (34%; *blue line*), those who were overall noncompliant (47%; *red line*), and those in whom compliance was nonevaluable (19%; *green line*). The nonevaluable EIG participants are shown to have consumption rates similar to those of the noncompliant EIG participants.

For the non–compliance-evaluable EIG participants, it is possible to look at individual interim questionnaire responses to assess how much of each allergenic food they were consuming for the questionnaires that were completed (see Fig E2 in this article's Online Repository at www.jacionline.org). This clearly indicates that allergenic food consumption levels in the nonevaluable children were similar to those in the noncompliant EIG participants.

### Food-specific per-protocol compliance

Food-specific per-protocol compliance in the EIG reflected the relative ease of the introduction of the different foods observed in Fig 4, and the results for the compliance-evaluable children were as follows: milk, 84% (451/537); peanut, 61% (336/549); egg, 42% (234/551); sesame, 52% (288/550); whitefish, 59% (318/543); and wheat, 39% (216/553). As a percentage of the whole EIG group (n = 652), these figures represent the following: milk, 69%; peanut, 52%; egg, 36%; sesame, 44%; whitefish, 49%; and wheat, 33%.

By 6 months of age, the per-protocol consumption target of 3 g of allergenic food protein per week was being by approximately 60% of EIG participants for egg and sesame, 75% for peanut and whitefish, 80% for wheat, and 90% for cow's milk (Fig 4).

**Fig E3:**
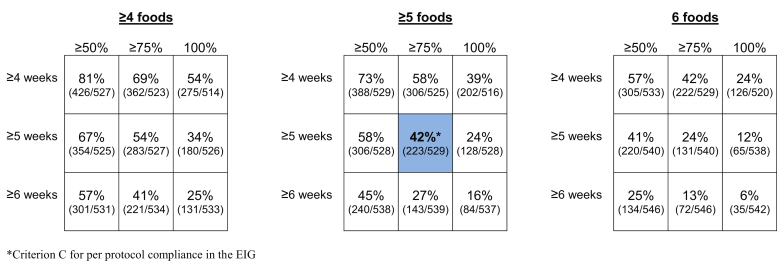
Influence of the number of foods consumed and the quantity and frequency of consumption on compliance in the compliance-evaluable EIG participants.

**Fig E4:**
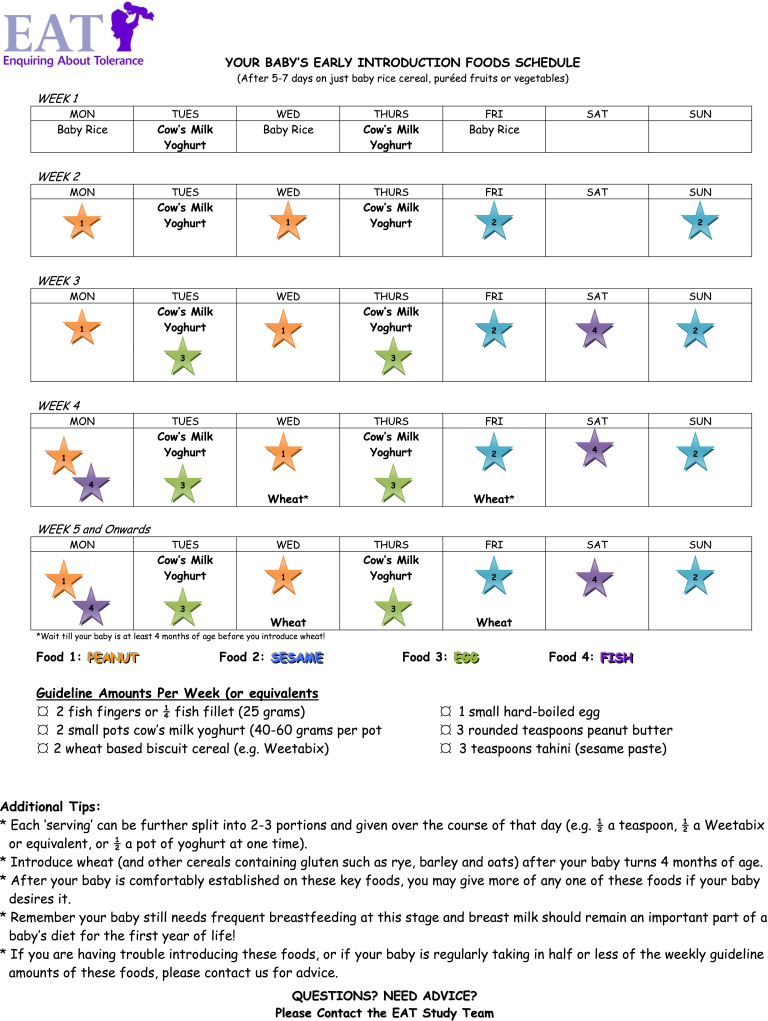
Example of an EIG allergenic food introduction regimen.

The effect of altering the number of foods eaten, both quantity and frequency, during this period is shown in Fig E3 in this article's Online Repository at www.jacionline.org. Compliance with the different permutations ranged from 6% to 81% depending on the stringency of the criteria used.

### Safety

Stopping rules for the study are shown in Table E2 in this article's Online Repository at www.jacionline.org. Detailed safety analyses will be reported in the primary outcome paper of the EAT study: however, stopping the study was not considered at any time point for safety reasons. The independent data monitoring committee did not raise any concerns regarding either group.

## Discussion

The infant diet in developed countries, such as the UK, is one in which consumption of many of the principal allergenic foods is minimal or absent during the first 6 months of life. Among 8- to 10-month-old infants in the IFS2010, egg and fish were being consumed less than once a week or never in 73% and 44% of infants, respectively.^14^ Remarkably, 45% of all mothers in the IFS2010 actively avoided giving at least 1 particular ingredient. The most common allergenic food avoided was as follows: nuts (peanuts and tree nuts), 41% of all mothers; eggs, 12%; dairy, 11%; fish/seafood, 8%; and gluten/wheat, 3%. Concern about allergies (36%) was the most common reason for avoidance overall, but this varied by food: egg, 40%; dairy, 47%; and nuts, 63%. Concern about the infant being too young for the food and the presence of eczema were also common reasons for avoidance.

However, there are countries in which early allergenic food exposure is different. Observational evidence has emerged from both developed countries, such as Israel,^19^ and developing countries, such as Ghana,^28^ where high amounts of peanut are consumed in a variety of forms during infancy, yet peanut allergy rates remain very low, suggesting a possible route of tolerance induction. Among Jewish children, genetic influences are not responsible because the prevalence of peanut allergy in Jewish children in the UK at 1.85% was significantly higher than the Israeli prevalence of 0.17%.^12^ It is interesting to note that the incidence of food allergy is believed to be increasing in Africa,^29^ and a delay in introduction and reduced quantity of consumption of peanut has been postulated as a possible cause.^30^, ^31^

Despite the fear of allergy expressed in the IFS2010 survey, particularly with regard to peanut, we have demonstrated that parents were prepared to introduce peanuts and other allergenic foods into their infant's diet at less than 6 months of age.

Comments on the ability of EIG families to fulfill the overall per-protocol compliance targets have to take account of the compliance status being nonevaluable for 19% of the EIG participants. Consumption data from the questionnaires that were completed in this group demonstrate that their consumption pattern was similar to the noncompliant EIG participants, and hence the true overall per-protocol compliance target in the EIG group was likely to have been closer to 34% than 42%.

This difficulty in achieving the overall per-protocol target of 5 or more foods at 3 g of allergenic protein or more per week for 5 or more weeks was not a clear dichotomy of no consumption versus per-protocol target consumption because we have demonstrated that among EIG families completing the 6-month questionnaire, the percentage who had never tried each of the allergenic foods was minimal. However, clearly for at least 58% of EIG participants, the amount consumed during this early period was less than the overall per-protocol target we had set. For 4 foods at 5 months of age and 2 foods at 6 months of age, 25% of EIG participants were not consuming the foods twice weekly, as requested, making it significantly harder to achieve the per-protocol target in only 1 meal per week (Fig 5). However, the proportion of EIG participants not reaching the 3-g per week per-protocol target by 6 months was greater than 25% for egg and sesame, suggesting that although once-weekly consumption might partly explain why 58% did not meet the target, for other EIG participants, the amount being consumed at their 2 (or more) weekly meals clearly was not sufficient to meet the 3-g per-protocol target when the consumption for that week was combined.

Despite the low figure for overall EIG per-protocol compliance, at an individual-food level, for evaluable EIG participants, compliance with our per-protocol target varied from 42% for egg to 84% for milk. Wheat compliance was lower than that for egg but was distorted by the introduction regimen, which did not allow wheat introduction before 4 months, hence leaving less weeks available to achieve the target level of consumption by 6 months of age.

We deliberately set the bar high for overall per-protocol compliance in the EIG because the amount of allergen protein needed to potentially induce oral tolerance is unknown. We wanted to ensure that the majority of those not meeting the 3-g per-protocol weekly target were still consuming allergenic food protein in a quantity that might induce tolerance (1 g of peanut protein twice weekly in our previous research).^12^ Our weekly per-protocol target had to balance the need to be recommending portion sizes appropriate for young infants with a frequency of consumption that was manageable for families given 6 foods were being introduced. Eighty-one percent of compliance-evaluable EIG children were consuming at least 2 g of protein a week (1 g of protein twice weekly) from at least 4 allergenic foods for at least 4 weeks between 4 and 6 months of age (see Fig E3).

Although overall compliance with the UK breastfeeding recommendations remains poor, the IFS2010 showed a continued increase in exclusive breastfeeding in the UK, with 69% of mothers exclusively breastfeeding at birth, up from 65% in 2005.^32^ Exclusive breastfeeding until 6 months of age remains rare, with only 1% achieving this, but rates of nonexclusive breastfeeding have increased from 25% at 6 months in 2005 to 34% in 2010. Within this context, the breastfeeding performance in the EAT study exceeded that observed in equivalent mothers in the IFS2010 at every time point, demonstrating the commitment of the participants and the study team to promote breast-feeding. For the first time in a randomized trial, our study demonstrates that early solid food introduction has no deleterious effect on breastfeeding duration, which is consistent with the findings in the observational study by Hörnell et al.^33^ This is particularly important because murine research has suggested that breastfeeding might be a vital component in the mechanism to induce tolerance in patients with allergic disease,^34^ and therefore the fact that 97% of EIG mothers continued to breastfeed while introducing allergenic foods might be a key part of our study findings.

The cohort's atopy status, as one would anticipate from the nature of the study, was enriched.^35^ Eighty-two percent of EAT participants had a parental history of atopy (mother and/or father with self-reported asthma, eczema, or hay fever) that was greater than the 51% rate of allergy (the above conditions and self-reported food allergy in either parent or a sibling) reported in the IFS2010. In the latter the rate in mothers with a managerial/professional occupation (more similar to EAT mothers) was 56%, which was still significantly less than in EAT. At the 3-year visit, EAT parents undergo SPTs to a panel of airborne allergens, as well as to any food to which the parent suspects they are allergic. This will allow an objective measure of the degree of atopy in EAT parents and the extent to which this corresponds with the high parent-reported atopy rate. Our filaggrin mutation inheritance rate (11.9%) was slightly higher than that observed in the Isle of Wight cohort study (10.3%)^36^ and a recent Irish birth cohort study (10.5%).^25^ Studies assessing unselected cohorts of 3-month-old infants are rare. The EAT visible eczema rate at age 3 months (24.4%) was higher than in the 6-month-old infants examined in the Irish cohort study (18.7%) by using the same diagnostic criteria, although the mean SCORAD score among those with eczema was significantly higher in the Irish study than in our study. The sensitization rate in the EIG in the EAT study was higher than the 1.2% observation in the Danish Allergy Research Centre cohort,^26^ but the latter only tested for 2 foods, milk and egg, and used only a commercial SPT solution for the latter.

The EAT study differs from the LEAP study in a number of important ways. First, the EAT study is conducted on unselected infants, whereas the LEAP study only studied high-risk infants with severe eczema, egg allergy, or both. Second, the EAT EIG receives multiple food allergens, as opposed to only peanut. Third, the EAT study introduces complementary feeding earlier from 3 months of age. It is noteworthy that the window of opportunity to induce tolerance to peanut might be narrow. In the LEAP screening study a significant number of infants with severe eczema, egg allergy, or both could not enter the study or adhere to the study protocol because of potential or proved pre-existing peanut allergy (SPT >4 mm and those infants who reacted at baseline).^17^ The possibility of earlier introduction of peanut (as early as 3 months of age) could potentially enhance prevention of peanut allergy in the general population by inducing tolerance in those children who would otherwise have peanut allergy early in the first year of life. It remains unknown whether the window of opportunity to induce tolerance varies by food. Observational studies have suggested a protective effect of introducing egg between 4 and 6 months of age^13^ and for introducing cow's milk protein–based formula milk before 14 days of age.^11^ Among the randomized controlled trials published thus far, the Solids Timing for Allergy Research study introduced egg to 4-month-old infants with a nonsignificant reduction in egg allergy incidence,^16^ and the LEAP study achieved peanut tolerance with introduction between 4 and 10 months of age.^18^

The EAT study has created 2 groups with significantly different early allergenic food exposure. This has been achieved without any adverse influence on breastfeeding performance. Although compliance with the early introduction of multiple foods in the amounts recommended proved difficult, at an individual food level, early introduction was more favorable. The EAT study design will allow us to assess the relative importance of the quantity, frequency, and number of allergenic foods in influencing food allergy development. We will also be able to investigate whether factors exist that can predict the likelihood of complying with the recommended EIG regimen. These findings will help inform future guidelines regarding early infant feeding policy.

**Clinical implications**

The EAT study demonstrates that multiple allergenic foods can be introduced into the infant diet. The introduction of allergenic foods was safe, and there was no adverse influence on breastfeeding.

## Figures and Tables

**Table E1:** Definition of IgE-mediated food allergy for the primary end point

Primary end point definition: Period prevalence of IgE-mediated food allergy to the 6 intervention foods between 1 and 3 years of age in both arms • Category 1A: Positive DBPCFC result at 1 or 3 years of age in a child sensitized to 1 of the 6 intervention foods • Category 1B: Positive DBPCFC result between 1 and 3 years of age in a child attending an unscheduled clinic visit who was child sensitized to 1 of the 6 intervention foods
Although the first 2 categories relate to events at between 1 and 3 years of age, we will include children potentially outside of this range in 2 exceptional circumstances:
• Category 2: Positive challenge (open or DBPCFC) result at between 6 months and 1 year of age that occurs in a child sensitized to 1 of the 6 intervention foods who subsequently refuses a DBPCFC at 1 and 3 years of age
Rationale: Before 6 months of age, only intervention children have challenges, and therefore this category is restricted to those infants who are 6 months or older.
• Category 3: History of food-induced allergy in a child with an SPT ≥5 mm
Rationale: There will be a small number of children who have an immediate-type allergic reaction and are significantly sensitized whose parents refuse to allow them to undergo any further challenge.
The relative contributions of children to the final outcome in each of these 4 categories will be presented separately, as well as the overall cumulative figure.

*DBPCFC*, Double-blind, placebo-controlled food challenge.

**Table E2:** Stopping rules in the EAT study

**Enrollment in the trial will be stopped pending review if:** • any death occurs; • a participant is admitted to an intensive care unit for an adverse event related (NCI-CTCAE attribution of adverse events code 4 or 5) to study intervention; or • a participant randomly assigned to the allergenic food consumption group experiences life-threatening anaphylaxis during the first 3 weeks of administration of the allergenic food.
**Enrollment in the trial will be stopped pending review if any of the following occurs for the EIG:** • an analysis is performed when 100 such participants in the EIG have been followed for 3 months demonstrates that the lower bound of the 95% CI for the proportion of participants in the EIG with IgE-mediated food allergy, as determined in an unscheduled clinic visit, is greater than 6%; • an analysis is performed when 200 participants per group have been followed up to 12 months of age demonstrates that the rate of serious adverse events is significantly greater at the .05 significance level in the allergenic food consumption group than in the SIG; or • an analysis is performed when 10 such participants in the allergenic food consumption group have been followed until 12 months of age demonstrates that 4 or more participants have experienced a related (NCI-CTCAE attribution of adverse events code 4 or 5) serious adverse event.

*NCI-CTCAE*, National Cancer Institute–Common Terminology Criteria for Adverse Events.

**Table I:** Overall per-protocol compliance criteria in the EAT study

Compliance definitions	Compliance-evaluable children meeting compliance definitions
**SIG (n = 606/651 children compliance evaluable)**	
• Criterion A: Exclusive breastfeeding for ≥3 months (water and/or oral rehydration solution allowed)	100% (606/606) (A) 12.0% have had water by 3 months of age
• Criterion B: Continued breastfeeding up to 5 months of age	99.7% (604/606) (B)
• Criterion C: No consumption of peanut, egg, sesame, fish, or wheat before 5 months of age	97.4% (590/606) (C)
• Criterion D: No introduction of cow's milk formula (or goat's milk formula [or consumption of <300 mL/d]) between 3 and 6 months of age	(1) No formula before 6 months: 85.6% (519/606) (2) Consumption of less than 300 mL/d: 8.8% (53/606) (*median age of introduction of 22 wk*) (1) or (2): 94.4% (572/606) (D)
**Overall SIG per-protocol compliance (meets all criteria)**	**92.1% (558/606) (A, B, C, and D)**
**EIG (n = 529/652 children compliance evaluable)**	
• Criterion A: Exclusive breastfeeding for 3 months' duration (water and/or oral rehydration solution allowed)	100% (529/529) (A) 13.1% have had water by 3 months of age
• Criterion B: Continued breastfeeding up to 5 months of age	99.6% (527/529) (B)
• Criterion C: Consumption of ≥5 of the allergenic foods in at least 75% of the recommended amount (3 g of allergen protein/wk) for at least 5 wk between 3 and 6 months of age	42.3% (224/529) (C)
**Overall EIG per protocol compliance (meets all criteria)**	**42.2% (223/529) (A, B, and C)**

Compliance status was nonevaluable for 7% (45/651) of the SIG and 19% (123/652) of the EIG participants.

**Table II:** Demographics and clinical assessment at trial enrollment

	SIG (%), n/N	EIG (%), n/N	UK data (%)
No. in group	651	652	
Demographics			
Median age at enrolment (wk)	14.7 (n = 651 [range, 13.0-18.0])	14.7 (n = 652 [range, 12.9-18.0])	
Sex			
Male	52.1 (339/651)	48.2 (314/652)	51.3^23^
Female	47.9 (312/651)	51.8 (338/652)	48.7
Ethnicity			
White	84.0 (547/651)	85.4 (557/652)	87.1^14^
Black	2.9 (19/651)	3.4 (22/652)	3.6
Asian#	1.7 (11/651)	2.6 (17/652)	6.5
Chinese	0.5 (3/651)	1.2 (8/652)	1.2
Mixed	10.9 (71/651)	7.4 (48/652)	1.6
Home location			
Urban	77.4 (503/650)	77.3 (503/651)	81.5^23^
Rural (nonfarm)	20.3 (132/650)	19.5 (127/651)	17.6
Rural (farm)	2.3 (15/650)	3.2 (21/651)	0.9
Pet ownership	44.6 (290/650)	40.6 (264/651)	77.9∗^24^
Maternal education (age at completion)			
≤16	6.2 (40/650)	5.2 (34/652)	18.8^14^
17-18	13.7 (89/650)	12.7 (83/652)	28.9
>18	80.2 (521/650)	82.1 (535/652)	52.3
Smoking			
Maternal (in pregnancy)	3.9 (25/650)	3.2 (21/651)	11.5^14^
Maternal (postpartum)	3.1 (20/650)	3.4 (22/651)	13.3^14^
Paternal	10.9 (71/650)	10.8 (70/651)	20.0^14^
Family history			
Median maternal age (y)	33 (n = 650 [range, 19-46])	33.5 (n = 652 [range, 19-45])	49% ≥30^14^
Siblings			
0	38.3 (249/651)	37.3 (243/652)	49.9^14^
1	36.9 (240/651)	39.3 (256/652)	33.5
2	16.4 (107/651)	14.9 (97/652)	10.9
≥3	8.5 (55/651)	8.6 (56/652)	5.0
Birth history			
Birth weight (g), mean (SD)	3560 (487 [n = 651])	3570 (489 [n = 651])	3489 (512 [Ireland]^25^)
Mode of delivery			
Vaginal	77.3 (503/651)	72.4 (472/652)	76.2^14^
Cesarean	22.7 (148/651)	27.6 (180/652)	24.8
Mean gestational age (wk)	39.7 (n = 651)	39.9 (n = 652)	
Participant enrollment atopy status			
Sensitization (SPT >0 mm)	NA	5.1 (33/652)	1.2† (Denmark)^26^
Filaggrin mutation	11.5 (69/598)	12.2 (74/608)	10.5‡ (Ireland)^25^
Visible eczema	24.2 (157/650)	24.5 (160/652)	18.7 (Ireland)§^25^
Median SCORAD score (infants with eczema)	7.5 (n = 157 [range, 3.5-49.2])	7.5 (n = 160 [range, 3.5-75.0])	21.5‖ (Ireland§^25^ [range, 0-88])
EIG median age of allergenic food first consumption (wk)			
Dairy	—	17.3	
Peanut	—	19.6	
Egg	—	19.6	
Sesame	—	19.6	
Whitefish	—	19.6	
Wheat	—	20.6	
Family atopy status (self-reported)			
Maternal			
Eczema	34.2 (222/650)	34.9 (227/651)	19.9^21^
Asthma	26.8 (174/650)	25.8 (168/651)	13.0^21^
Hay fever	46.9 (305/650)	43.8 (285/651)	25.2^21^
Food allergy	16.9 (110/650)	21.8 (142/651)	27.5^27^
Maternal atopy (eczema, asthma, or hay fever)	63.2 (411/650)	61.9 (403/651)	40.8^21^
Maternal atopy (eczema, asthma, hay fever, or food allergy)	66.2 (430/650)	65.8 (428/651)	
Paternal			
Eczema	21.1 (137/650)	18.9 (123/651)	8.4^21^
Asthma	23.5 (153/650)	21.8 (142/651)	12.0^21^
Hay fever	41.1 (267/650)	40.3 (262/651)	20.7^21^
Food allergy	10.0 (65/650)	11.2 (73/651)	14.0^27^
Paternal atopy (eczema, asthma, or hay fever)	55.7 (362/650)	50.5 (329/651)	30.4^21^
Paternal atopy (eczema, asthma, hay fever, or food allergy)	57.1 (371/650)	52.8 (344/651)	
Parental			
Parental atopy (eczema, asthma, or hay fever)	83.9 (545/650)	80.0 (521/651)	57.7^21^
Parental atopy (eczema, asthma, hay fever, or food allergy)	85.4 (555/650)	82.5 (537/651)	51.0^14^¶
Maternal allergenic food consumption			
During pregnancy	100.0 (639/639)	100.0 (631/631)	
During breastfeeding	100.0 (639/639)	100.0 (631/631)	

UK data were used for comparison unless a suitable equivalent study was not available.

*NA*, Not applicable.

∗ Pet ownership at less than 3 years of age.

† Denmark: 3 months of age—cows’ milk (0.6%) and hen's egg (0.6% [commercial SPT solutions]) and fresh cows' milk (0.6%). A positive SPT response was defined as a mean wheal size of 2 mm or greater than that elicited by the negative control.

‡ Four filaggrin mutations were assessed: R501X, 2282Del4, S3247X, and R2447X.

§ Ireland: 6 months of age.

‖ Mean SCORAD score.

¶ Parental and/or sibling (eczema, asthma, hay fever, or food allergy).

# Asian refers to Indian, Pakistani, and Bangladeshi.

## Methods

### EIG introduction regimen

After normal blood test results at enrollment (complete blood count, bone, liver, renal, and lipid profile tests), EIG infants proceeded to introduce baby rice, pureed fruits or vegetables, or both during the first week until they were established on solid food. They then continued with these solids and additionally introduced cow's milk yogurt on 2 days of the second week. During weeks 3 and 4 of the early introduction regimen, peanut, hard-boiled egg, sesame, and whitefish (cod) were introduced sequentially in a random order, with 2 new foods introduced per week. Finally, wheat was introduced in week 5, reflecting the guidance on optimal timing of wheat introduction after 4 months of age,^E1^ and by week 6, infants were ideally consuming the required amount of all 6 allergenic foods each week (Fig E4).

Introduction of nonallergenic foods was not restricted during this process, and the allergenic foods could be given in combination with other foods or each other once the allergenic foods had been successfully introduced and well tolerated.

The exact volume of allergen protein necessary to induce oral tolerance is unknown, but participants were asked to consume the equivalent of 2 g of each allergenic food protein twice each week (4 g of allergen protein per food per week). Therefore the full weekly amount for the allergenic foods consisted of 2 small 40- to 60-g portions of cow's milk yogurt, 3 rounded teaspoons of peanut butter, 1 small hard-boiled egg (<53 g), 3 rounded teaspoons of sesame paste, 25 g of whitefish, and 2 wheat-based cereal biscuits (eg, Weetabix).

### Allergenic protein dose

The 2 g of allergen protein per dose is based on our earlier research investigating peanut consumption among Israeli and UK Jewish children.^E2^ In that study the median frequency of peanut consumption was 8 times per month (ie, twice per week), and the median monthly consumption amount was 7.1 g (ie, about 1 g per dose). Although the Jewish infants were older than those in our study (8-14 months), research also showed that significant numbers were eating much bigger doses than the 1-g average.

Therefore we elected to adopt a twice-weekly consumption regimen per food in accordance with this earlier research. We set our dose target at 2 g. This allowed for infants falling short of this target to still achieve a level of consumption that our earlier research had shown appeared to be tolerance inducing.

### Per-protocol compliance status: Definitions

#### SIG overall per-protocol compliance criteria

For the SIG, the criteria in Table I reflect a pragmatic interpretation of the current UK status quo: an incomplete following of the infant feeding recommendations. Thus allergenic food introduction from 5 months (criterion C), by which point 75% of mothers in IFS2010 have introduced solids to their baby, including rusks (hard, dry infant biscuits) and yogurts, as well as up to 300 mL/d cow's milk formula consumption after enrollment (criterion D), was considered acceptable. The volume was chosen such that the majority of milk consumed by the infant was still breast milk. The amount of breast milk consumed by exclusively breastfed infants was evaluated in a recent systematic review.^E3^ At 3 to 4 months of age, the mean transfer volume of breast milk was 779 g/d (SD, 40 g/d); at 5 months, it was 827 g/d (SD, 39 g/d); and at 6 months, it was 894 g/d (SD, 87 g/d).

#### EIG overall per-protocol compliance criteria

For the EIG, criterion C aimed for consumption of at least 5 of the 6 allergenic foods in at least 75% of the 4-g recommended amount (3 g of allergen protein per food per week) for at least 5 individual weeks between 3 and 6 months of age.

#### EIG food-specific per-protocol compliance criteria

In the EIG food-specific per-protocol compliance was determined by using the same threshold as criterion C: consumption of a specific allergenic food in at least 75% of the recommended amount (3 g of allergen protein/wk) for at least 5 weeks between 3 and 6 months of age.

### Per-protocol compliance status: Appraisal

Criterion A for both groups, exclusive breastfeeding at the point of enrollment, was a prerequisite to taking part in the study, and hence compliance with this was complete. The ability to determine compliance with the other individual criteria relied on data being available from the online questionnaires.

#### SIG: Overall per-protocol compliance

For the SIG, criterion B (continued breastfeeding up to 5 months) and criterion D (no or minimal introduction of cow's milk formula between 3 and 6 months of age) could be determined from any of the interim questionnaires because every questionnaire asked at what age the participant stopped breastfeeding and at what age they started giving their child formula milk. Criterion C (no consumption of peanut, egg, sesame, fish, or wheat before age 5 months) required the SIG mother to have completed both the 4- and the 5-month interim questionnaires for their compliance with this criterion to be evaluable.

#### EIG: Overall per-protocol compliance

For the EIG, criterion B was the same as for the SIG and hence could be determined from the response to any interim questionnaire. However, it was criterion C that made assessment of compliance in the EIG more difficult to be achieved than in the SIG. Criterion C was consumption of at least 5 of the allergenic foods in at least 75% of the recommended amount (3 g of allergen protein/wk) for at least 5 weeks between 3 and 6 months of age. For this to be determined, the EIG family needed to have completed all 3 of the 4-, 5-, and 6-month interim questionnaires.

### Sensitization status

At the enrollment visit, EIG infants underwent SPTs in duplicate to whole foods for fresh cow's milk, raw egg white, and tahini (sesame paste) and to cod, wheat, and peanut by using commercial solutions (Stallergenes, Didcot, United Kingdom). At 1 year of age, SPTs were undertaken for the whole cohort with commercial solutions only to a panel of foods (the 6 intervention foods, soya, and kiwi) and aeroallergens (house dust mite, cat, dog, 6-grass pollen mix, and 3-tree pollen mix). At 3 years of age, the 1-year investigations were repeated with additional SPTs to commercial solutions of Brazil nut, hazel nut, cashew, almond, and walnut and also to salmonella-free raw egg white. SPT wheal size was the mean wheal size of the duplicate tests. Attending parents underwent SPTs and blood tests at the 3 year visit.

### Food allergy status

All participants underwent an assessment of their allergy status at 1 and 3 years of age with SPTs to all 6 study foods as above and double-blind, placebo-controlled food challenges where sensitized (SPT >0 mm) and not consuming the food regularly or open challenges if they fulfilled the study's frequent consumer criteria (consumption of an EAT portion [2 g of protein] in the last month and ≥3 times ever). Any participants with a positive open challenge result then underwent a double-blind challenge. At any time during the study, parents could report any allergy symptoms, in which case an unscheduled clinic visit with skin prick testing and food challenge, if indicated, was undertaken. The primary outcome, IgE-mediated (positive SPT response) allergy to 1 or more of the 6 early introduction foods, will be determined as outlined in Table E1.

### Skin assessment

All children were examined for eczema by using the UK diagnostic criteria–based photographic protocol of the International Study of Asthma and Allergies in Childhood Phase 2.^E4^ Disease severity was determined by using the SCORAD index,^E5^ and measurement of transepidermal water loss was done by using the Biox AquaFlux AF200 closed condenser chamber device (Biox Systems Ltd, London, United Kingdom) on the unaffected skin of the volar aspect of the forearm.^E6^

### Microbiome

The infant microbiome was assessed through collection of skin swabs at 3 and 12 months and stool samples at 3, 5, and 12 months of age.

### Dust samples

To corroborate reported consumption of egg and peanut, dust samples were collected from the infants' beds at enrollment and at 1 year of age to measure egg and peanut protein levels in dust by means of ELISA.^E7^ Measurement of peanut in household dust has been used to assess the infants' consumption of peanut because the levels of this protein in dust have been shown to correlate well with results on a food frequency questionnaire for consumption of peanut^E8^ and were also used to corroborate peanut consumption in the LEAP study.^E9^

### Growth

A broad panel of anthropometric measurements were made, including length/height, weight, head circumference, triceps and subscapular skin fold thickness, and mid-upper arm circumference.

### Blood sampling

Venous blood was obtained at the 3 visits. The enrollment sample was screened for the 6 most common *FLG* mutations (R501X, 2282del4, R2447X, S3247X, 3673delC, and 3702delG).^E10^ After safety blood measurements (full blood count, renal and liver function, and bone metabolism), spare serum was stored (−70°C) for determination of levels of specific IgE to the allergic foods and component-resolved diagnostics to peanut. PBMCs were stored in liquid nitrogen for subsequent lymphocyte studies.

### Food diary

At 6, 12, and 36 months of age, parents completed a 5-day food diary, recording in detail their child's diet, including portion sizes, ingredients, and commercial food brands, to allow a full nutritional breakdown of macronutrients during the trial.
